# Hypothetical Protein BPSL3393 of *Burkholderia pseudomallei* is Involved in Ethanolamine Catabolism

**DOI:** 10.21315/tlsr2017.28.2.5

**Published:** 2017-07-31

**Authors:** Ooi Gim Luan, Hokchai Yam, Razip Samian, Mustafa Fadzil Farid Wajidi, Nor Muhammad Mahadi, Suriani Mohamad, Nazalan Najimudin

**Affiliations:** 1 School of Biological Sciences, Universiti Sains Malaysia, 11800 USM Pulau Pinang, Malaysia; 2 School of Distance Education, Universiti Sains Malaysia, 11800 USM Pulau Pinang, Malaysia; 3 Malaysia Genome Institute, Jalan Bangi, 43000 Kajang, Selangor, Malaysia; 4 School of Pharmaceutical Sciences, Universiti Sains Malaysia, 11800 USM Pulau Pinang, Malaysia; 5 Faculty of Applied Sciences, UCSI University, Jalan Menara Gading, 56000 Kuala Lumpur, Malaysia

**Keywords:** *Burkholderia pseudomallei*, BPSL3393, ethanolamine

## Abstract

*Burkholderia pseudomallei* is a soil-dwelling bacterium that causes a globally emerging disease called melioidosis. Approximately one third of the in silico annotated genes in its genome are classified as hypothetical genes. This group of genes is difficult to be functionally characterised partly due to the absence of noticeable phenotypes under conventional laboratory settings. A bioinformatic survey of hypothetical genes revealed a gene designated as BPSL3393 that putatively encodes a small protein of 11 kDA with a CoA binding domain. BPSL3393 is conserved in all the *B. pseudomallei* genomes as well as various in other species within the genus *Burkholderia*. Taking into consideration that CoA plays a ubiquitous metabolic role in all life forms, characterisation of BPSL3393 may uncover a previously over-looked metabolic feature of *B. pseudomallei*. The gene was deleted from the genome using a double homologous recombination approach yielding a null mutant. The BPSL3393 mutant showed no difference in growth rate with the wild type under rich and minimal growth conditions. An extensive metabolic phenotyping test was performed involving 95 metabolic substrates. The deletion mutant of BPSL3393 was severely impaired in its ethanolamine metabolism. The growth rate of the mutant was attenuated when ethanolamine was used as the sole carbon source. A transcriptional analysis of the ethanolamine metabolism genes showed that they were down-regulated in the BPSL3393 mutant. This seemed to suggest that BPSL3393 functions as a positive regulator for ethanolamine metabolism.

## Abstract (ru)

*Burkholderia pseudomallei* adalah bakterium kediaman tanah yang menyebabkan penyakit muncul secara global yang dikenali sebagai melioidosis. Dianggarkan satu pertiga daripada gen siliko di dalam genom dikelaskan sebagai gen hipotesis. Kumpulan gen ini sukar untuk dicirikan secara fungsional, sebahagiannya disebabkan oleh ketiadaan fenotip yang ketara di bawah tetapan makmal konvensional. Kajian bioinformatik gen hipotetikal menunjukkan gen yang ditetapkan sebagai BPSL3393 yang menguraikan protein kecil 11 kDA dengan domain mengikat CoA. BPSL3393 dipelihara dalam semua genom *B. pseudomallei* serta pelbagai spesies lain dalam genus *Burkholderia*. Dengan mengambil kira bahawa CoA memainkan peranan metabolik di mana-mana dalam semua bentuk kehidupan, pencirian BPSL3393 mungkin mendedahkan ciri metabolik *B. Pseudomallei* yang sebelum ini terlepas pandang. Gen ini dipadamkan dari genom menggunakan pendekatan penggabungan semula homolog berganda yang menghasilkan mutan null. Mutan BPSL3393 tidak menunjukkan perbezaan kadar pertumbuhan dengan jenis yang liar di bawah keadaan pertumbuhan yang kaya dan minima. Ujian fenotip metabolik yang meluas dilakukan dengan melibatkan 95 substrat metabolik. Pemotongan mutan BPSL3393 teruk terjejas dalam metabolisme etanolaminanya. Kadar pertumbuhan mutan itu dilemahkan apabila etanolamina digunakan sebagai sumber karbon tunggal. Analisis transkrip gen metabolisme etanolamina menunjukkan bahawa mereka telah dikawal dalam mutan BPSL3393. Ini seolah-olah menunjukkan bahawa fungsi BPSL3393 sebagai pengawal selia positif untuk metabolisme etanolamina.

## INTRODUCTION

*Burkholderia pseudomallei*, the causal agent of melioidosis, posts a potential threat to human health. Melioidosis is endemic in Southeast Asia and Northern Australia and has expanded to other continents during the past decade (Currie *et al.* 2008). There are various clinical manifestations of melioidosis ranging from acute to chronic with either a localised or systemic infection (Renella *et al.* 2006). The most common clinical observation of melioidosis is septicaemia with bacterial dissemination to distant organs such as spleen, liver and lung causing abscess (Stanton *et al.* 1924). The genome of *B. pseudomallei* K96243 comprises two circular chromosomes of 4.07 Mb and 3.17 Mb, respectively (Holden *et al.* 2004). Approximately 34% of the predicted genes were annotated as functionally uncharacterized hypothetical genes. These genes might contribute to the growth and survival of *B. pseudomallei* or they could be involved in obscure biological processes that yet to be uncovered. Functional characterisations of this group of genes will give a better understanding on the biology of *B. pseudomallei.* A bioinformatics survey of these hypothetical genes revealed an interesting hypothetical gene BPSL3393. Its gene product is predicted to be 11 kDa and contains a Coenzyme A (CoA) binding domain which indicates that it might play a role in a CoA related metabolism.

CoA is an essential cofactor which plays a crucial role in cell metabolism either as an acyl group carrier or a carbonyl-activating group. The most common CoA intermediate is in the form of acetyl-CoA which is involved in several vital pathways such as oxidative decarboxylation of pyruvate, catabolism and synthesis of amino acids, β-oxidation and synthesis of fatty acids and the tricarboxylic acid (TCA) cycle (Lipmann 1953).

In this study, a reverse genetic approach was applied to elucidate the biological function of BPSL3393. An unmarked deletion mutant was constructed via homologous recombination. Since the BPSL3393 gene might be involved in bacterial metabolism, the mutant was subjected to metabolic phenotyping. A total of 95 carbon metabolites were tested using Biolog GN2 MicroPlate™. The mutant showed reduced utilisation rate when ethanolamine was used as the carbon source. Hence, a gene expression study was also performed to study the expression levels of ethanolamine catabolism genes. In addition, physiological characterisations such as microscopic analysis, growth kinetic studies and the effect of ethanolamine on bacterial growth were also conducted.

## MATERIALS AND METHODS

### Bacterial Strains, Plasmids and Cell Growth Conditions

Bacterial strains and vectors used in this study are listed in Table 1. *B. pseudomallei* K96243 and *E. coli* strains were grown either on Luria-Bertani (LB) or M9 minimal medium (Sambrook & Russell 2001) at 37°C. Bacterial strains harbouring plasmids pDM4 (Milton *et al.* 1996) or pUD3393 were grown in Blomfield medium (10 g bacto-tryptone, 5 g bacto-yeast extract, supplemented with 10% sucrose) (Blomfield *et al.* 1991). Antibiotics were supplemented with appropriate concentrations when required: 75 μg/mL ampicillin for selection of *E. coli* JM109 transformants harbouring pUD-RBC, 20 ug/mL chloramphenicol for selection of *E. coli* S17 transformants harbouring pUD3393, 150 μg/mL chloramphenicol and 25 μg/mL gentamicin were supplemented for selection of transconjucants.

### Construction of BPSL3393 Deletion Mutant

BPSL3393 deletion mutant was constructed via homologous recombination using plasmid pDM-4, a non-replicative plasmid in *B. pseudomallei* (Milton *et al.* 1996). All the primers used for the construction of the deletion alleles are listed in Table 2. Briefly, the upstream (US) and downstream (DS) flanking regions of BPSL3393 ORF of approximately 1 kb were individually PCR-amplified. The resultant PCR products were restricted with *Sph*I. Subsequently, the digested US and DS fragments were ligated and amplified with primers USF and DSR to produce a combined 2 kb fragment of contiguous US-DS flanking DNA. This fragment was initially cloned into the RBC TA cloning vector (RBC Bioscience, Taiwan) to generate pUD-RBC using *E. coli* JM109 as the cloning host. The USDS insert was then excised with the restriction enzymes *XhoI* and *SacI* and was then recloned into pDM4 using *E. coli* S17 as the host. The resultant plasmid was named pUD3393 and the DNA insert was verified by DNA sequencing using primers NQCAT and NQREV. Isolation of the mutant was performed accordingly to the protocols described by Brown *et al*. (2004). Briefly, the pUD3393 was conjugally transferred into *B. pseudomallei* K96243 and recombined into its chromosome. The resulting transconjugants were selected based on their resistance to 25 μg/mL gentamycin and 150 μg/mL chloramphenicol. They should be merodiploid possessing two copies of flanking regions due to the single-crossover event that inserted the entire plasmid into the chromosome at the homologous site. The cells were then selected on Blomfield medium without antibiotic and supplemented with 10% sucrose to allow second recombination event that resulted in the removal of plasmid backbone from the genome. The developed colonies were either BPSL3393 deletion mutant or wild type strain. PCR screening was performed to distinguish mutants from wild-type strain using a pair of outer primers (USF-EXT & DSR-EXT) which complement to the outer region of the US-DS homologous regions. The mutant should produce a PCR fragment of 2.1 kb due to the BPSL3393 gene deletion while the wild type strain should produce a longer fragment of 2.4 kb. The shorter DNA fragments amplified from the mutant candidates were subjected to DNA sequencing for verification of BPSL3393 deletion. The deletion mutant was labelled as ΔBPSL3393. The merodiploid and mutant cells were eradicated by autoclaving after all characterisations have been performed.

### Electron Microscopy

Both the wild-type and ΔBPSL3393 mutant strains were grown to exponential phase using LB broth or M9 minimal medium. A volume of 5 ml of overnight culture was harvested at 4800 g for 10 min. The processing protocols of scanning electron microscopy (SEM) and transmission electron microscopy (TEM) were previously described in (Nation 1983) and (Skepper 1999), respectively. The SEM samples were examined by using the Leo Supra50 VP Field Emission scanning electron microscope (Carl-Zeiss SMT, Oberkochen, Germany) equipped with Oxford NCA 400 energy dispersion X-ray microanalysis system (Oxford Instruments, Bucks, UK). The TEM samples were examined by using the Philips CM12 (Philips Electron Optics, Eindhoven, Netherlands).

### Analysis of Cell Growth

Both wild-type and ΔBPSL3393 mutant strains were individually cultured in 20 mL LB medium and M9 minimal medium, respectively. The cultures were incubated at 37°C, 180 rpm for 18 h. The overnight cultures were used as the starting cultures for the growth curve analysis. The OD_595_ of overnight cultures were measured and seeded to a fresh 500 mL medium to the initial cell density of OD_595_ 0.05. The cultures were cultured at 37°C, 180 rpm and the OD_595_ readings were determined every 2 h for up to 48 h. Each strain was analysed in triplicate.

### Phenotype MicroArray Analysis

A high throughput phenotypic study was performed on the mutant and wild type strains to test their ability to consume a wide range of metabolites using the 96-well GN2 MicroPlate™ sytem (Biolog Inc., US). The Phenotype MicroArray (PM) analysis was performed according to Bochner *et al*. (2001). Colonies of wild type and mutant strains grown overnight on Luria agar (LA) were inoculated into GN/GP-IF inoculating fluid (Biolog Inc., US) to achieve a cell density corresponding to 50% transmittance in a Biolog turbidimeter. The suspension was transferred into the 96-wells GN2 MicroPlate™ with each well containing 150 μl of suspended cells. The GN2 MicroPlate™ was incubated at 30°C in the Omnilog^R^ PM reader system (Biolog Inc., US) and changes of colour in the wells were monitored automatically every 15 min for 96 h. Each strain was analysed in triplicate by using three individual plates. The Biolog GN2 MicroPlate contained a total of 95 metabolites and the metabolism rate of each metabolite was deduced based on the colour change over incubation time.

### Analysis of Cell Growth in Ethanolamine Minimal Medium

Based on the results of the PM analysis, the *B. pseudomallei* ΔBPSL3393 mutant showed that the utilisation rate of ethanolamine was reduced compared to the wild type strain. Ethanolamine can be utilised by bacteria as a carbon or nitrogen source. Hence, an analysis of growth was performed using a modified M9 minimal medium supplemented with ethanolamine in replacement to glucose. The ethanolamine minimal medium consisted of the following ingredients: KH_2_PO_4_ (15 g/l), Na_2_HPO_4_ (33.89 g/l), NaCl (2.5 g/l), 0.1 mM CaCl_2_, 2 mM MgSO_4_ and 200 nM vitamin B12 and 82 mM ethanolamine hydrochloride. To elucidate the ability of *B. pseudomallei* to utilise ethanolamine as the sole carbon source, 5 g/l of NH_4_Cl was supplemented into the medium. Similarly, 0.4% glucose was added to the medium to elucidate the ability of *B. pseudomallei* to utilise ethanolamine as the sole nitrogen source. Both wild-type and mutant strains were cultured for 18 hours in 20 mL of LB medium, incubated at 37°C and agitated at 180 rpm, and these cultures were used as the inocula for the growth analysis. The overnight cultures were washed with phosphate buffer prior to seed a 500 mL fresh media to achieve initial cell density of OD_595_ 0.05. The cultures were incubated at 37°C, 180 rpm and the OD_595_ readings were taken every 4 h for a period of 10 days. Each strain was analysed in triplicate.

### RT-qPCR Analysis

Since the phenotypic microarray analysis revealed that ΔBPSL3393 mutant showed reduced ethanolamine utilisation, the expressions of the ethanolamine degradation-related genes were studied by using RT-qPCR. The list of genes are as shown in Table 3. Both wild-type and ΔBPSL3393 mutant strains were cultured in ethanolamine minimal medium for three days. The cells were harvested by centrifugation at 4800 g for 10 min at 4°C. The total RNA extraction was performed by using TRIzol reagent (Ambion) accordingly to the manufacturer’s protocol. The RNA samples were subjected to DNase treatment (Ambion) and were then purified using acidic phenol-chloroform prior to RT-qPCR analysis. The sequences of the primers are as listed in Table 3 and the housekeeping *dnaK* gene was used for data normalisation. The RT-qPCR reaction mixtures were prepared according to the manufacturer’s protocol of iScript™ One-Step RT-PCR (Bio-Rad, USA). The PCR parameter used for RT-qPCR study was 10 min at 50°C for cDNA synthesis, 5 min at 95°C for reverse transcriptase inactivation, followed by 30 cycles of 10 sec at 95°C and 30 sec at 55°C for PCR cycling, 10 sec at 95°C, 0.05 sec at 65°C and 10 sec at 95°C for melting curve. The relative gene expression levels of the ethanolamine related genes for wild type and mutant strains were calculated using the method as suggested by Schmittgen and Livak (2008). Each gene was analysed in triplicate for both strains. Student’s t-test was applied for the assessment of significant difference (*p* < 0.05) between wild type and mutant strains.

## RESULTS

### Construction of *B. pseudomallei* BPSL3393 Deletion Mutant

**Figure 1:**
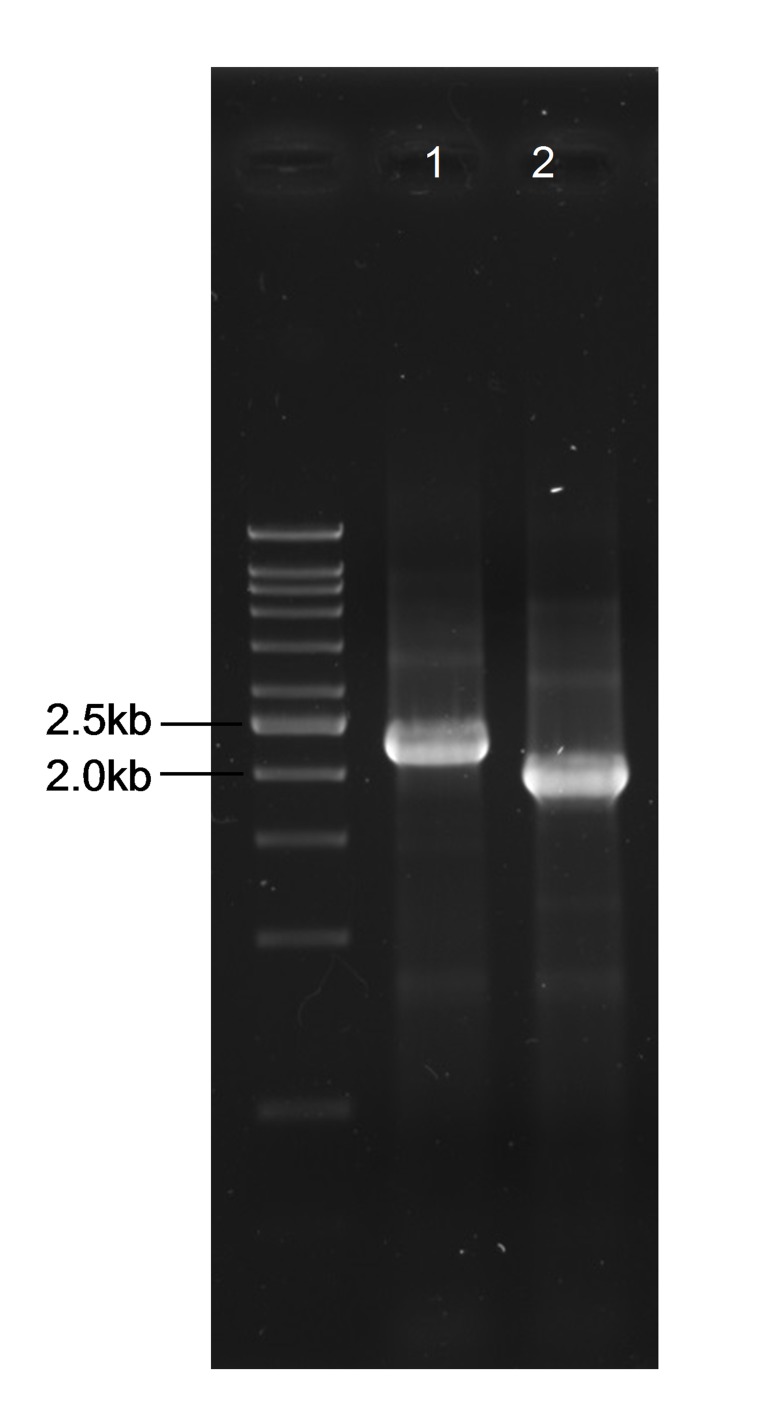
Markerless deletion of the *B. pseudomallei* BPSL3393 gene. Gel image of PCR products amplified from wild type strain (lane 1) and ΔBPSL3393 mutant (lane 2). The mutant gave shorter PCR amplicon compared to the wild type strain indicating the deletion of BPSL3393 gene.

The unmarked deletion mutant was constructed by homologous recombination and the putative mutant was verified by PCR amplification using a pair of external primers. The mutant gave a PCR amplicon of approximately 300 bp lesser than the wild-type as shown in Figure 1. DNA sequencing further confirm that the BPSL3393 had been deleted and the resultant mutant strain was named as ΔBPSL3393.

### Microscopic Observations Revealed That The ΔBPSL3393 Mutant Showed Similar Cell Characteristics to the Wild Type Strain

Electron microscopy analyses were performed to compare the cell surface and internal structures of the wild type and mutant strains. Both wild type and mutant strains did not show any significant difference in their surface morphology when grown in either LB or M9 minimal medium (image not shown). This suggested that the gene BPSL3393 was not involved in the determination of cellular morphology of *B. pseudomallei* K96243. Transmission electron microscopy revealed white globules resembling polyhydroxybutyrate (PHB) granules were observed within the cells of both strains. Both strains, grown in either LB or M9 minimal medium, showed a similar pattern of internal cell structure with no discernible difference observed (image not shown). These results concluded that deletion of BPSL3393 did not affect the interior and exterior configurations of the cells.

### The ΔBPSL3393 Mutant Cells Aggregated In M9 Minimal Medium

**Figure 2:**
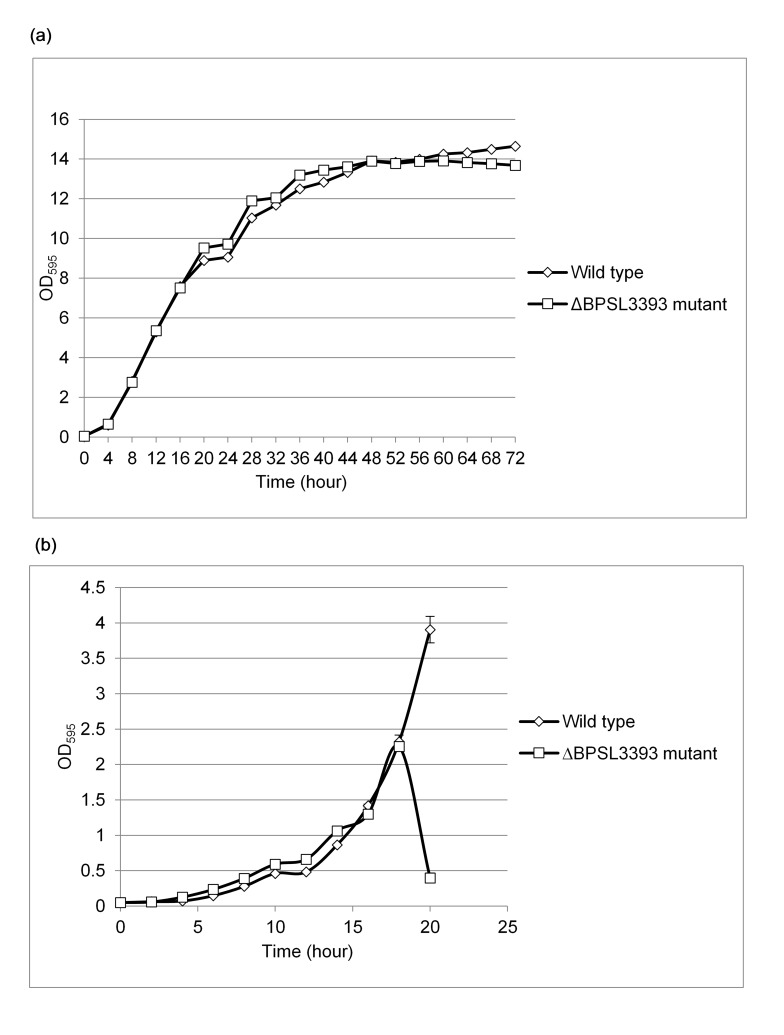
The growth curve analysis of *B. pseudomallei* ΔBPSL3393 mutant and wild type strain. (a) both strains have similar growth pattern in LB medium, where both strains reached plateu at approximately 28th hour. (b) *B. pseudomallei* ΔBPSL3393 mutant strain showed a drastically decrease in OD at 18th hour due to cells aggregation in M9 minimal medium. The error bars represent the values of standard errors.

The growth curves of the *B. pseudomallei* K96243 wild-type and ΔBPSL3393 mutant in LB and M9 media were determined and the results are shown in Figure 2. In LB medium, both wild-type and ΔBPSL3393 mutant showed similar growth rate. The exponential phase for both strains began after approximately 2 h and both strains reached stationary phase at approximately the 28th hour. In M9 minimal medium, the growth rates of both strains were slower as compared to the LB medium. Both strains entered the exponential phase after approximately 4 hours. However, at approximately 18th hour, the ΔBPSL3393 mutant cells started to aggregate while the wild type strain was still propagating. The aggregation appeared as insoluble clumps that settled to the bottom of the broth culture. Thus, the OD reading of the planktonic portion of the mutant culture dramatically dropped after 18th hour incubation. Therefore, BPSL3393 might play a role in maintaining the planktonic nature of the bacterial cells in limiting nutrient condition.

### The ΔBPSL3393 Mutant Showed Decreased Ethanolamine Utilisation Rate

**Figure 3:**
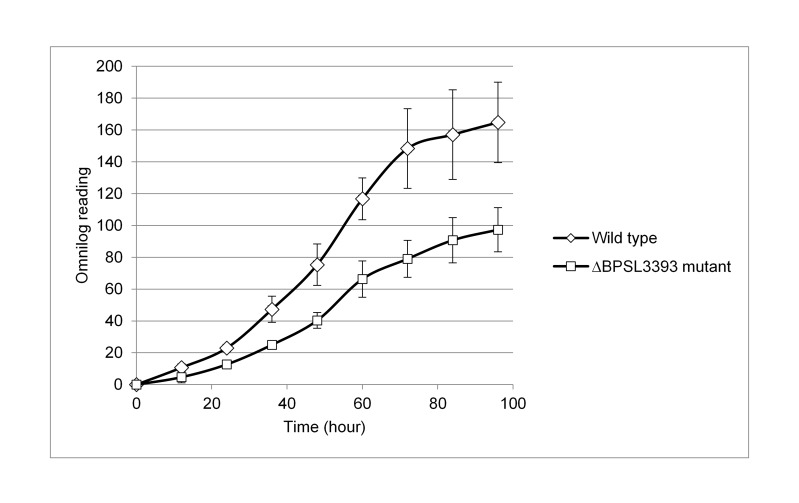
Graph of ethanolamine utilisation rate of *B. pseudomallei* ΔBPSL3393 mutant and wild type. *B. pseudomallei* ΔBPSL3393 mutant showed reduced utilisation ability as compared to wild type strain. The error bars represent the values of standard errors.

The Biolog GN2 MicroPlate™ was employed in this study to elucidate the metabolic role of BPSL3393. A total of 95 substrates were screened. The utilisation pattern did not show any significant difference between the mutant and wild type strains for all substrates except ethanolamine. The utilisation curve of ethanolamine is as shown in Figure 3. The ΔBPSL3393 mutant showed a decrease in ethanolamine utilisation. At the end of the 96th hour incubation, the ΔBPSL3393 mutant showed approximately 50% reduction in ethanolamine utilisation compared to the wild type. It is therefore evident that BPSL3393 plays a role in ethanolamine metabolism.

### ΔBPSL3393 Mutant and Wild Type Strains Showed Similar Growth Patterns in Ethanolamine Minimal Medium

**Figure 4:**
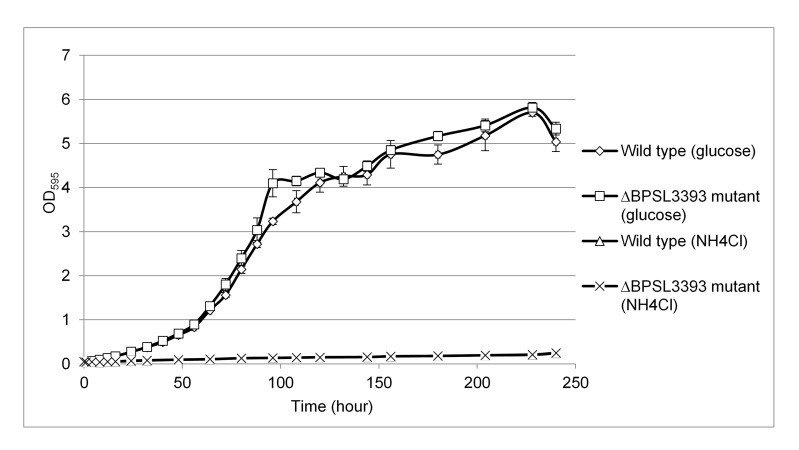
The growth curve analysis of *B. pseudomallei* ΔBPSL3393 mutant and wild type strain in ethanolamine supplemented media. The growth of *B. pseudomallei* ΔBPSL3393 mutant showed similar pattern with wild type in ethanolamine minimal medium supplemented with NH_4_Cl and ethanolamine minimal medium supplemented with glucose. *B. pseudomallei* showed much better cell growth when the ethanolamine minimal medium was supplemented with carbon source compared to nitrogen source. The error bars represent the values of standard errors.

The reduced rate of ethanolamine utilisation shown by the ΔBPSL3393 mutant could simply be due to a different cell growth rate compared to the wild type. However, growth analysis indicated that both ΔBPSL3393 mutant and wild type strains had similar growth rate in both ethanolamine minimal media (Figure 4). Therefore, differential growth rates are not a factor in the different readings of the wild type and ΔBPSL3393 mutant in the ethanolamine utilisation assay. A much better cell growth was observed when the ethanolamine minimal medium was supplemented with carbon source compared to nitrogen source. Both wild type and mutant cells achieved optical density values of higher than 5 after 240 h incubation in glucose supplemented ethanolamine minimal medium. However, cell densities of both strains were below 0.3 after same period of incubation in NH4Cl supplemented ethanolamine minimal medium. This observation indicated that ethanolamine is more favourably consumed as nitrogen source by *B. pseudomallei* K96243.

### Ethanolamine Catabolism Genes Were Down-regulated in ΔBPSL3393 Mutant

**Figure 5:**
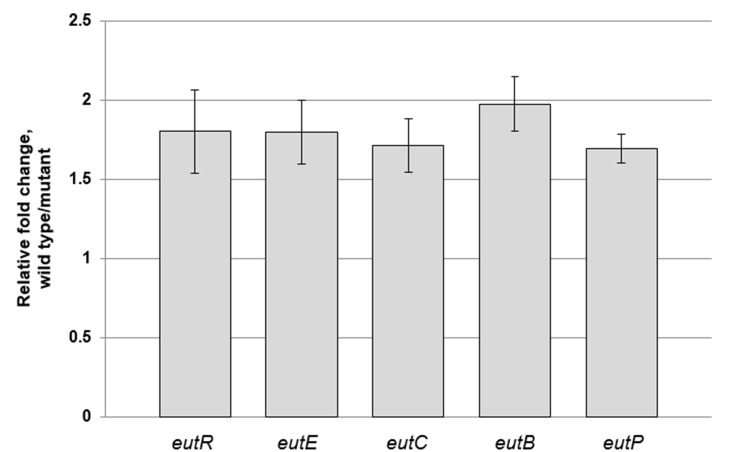
Result of *eut* genes expression study. The *eut* genes in the ΔBPSL3393 mutant were down-regulated compared to the wild type strain. The relative fold change values were at least 1.5 for each of the *eut* genes examined. The error bars represent the values of standard errors.

The relative fold changes for ethanolamine catabolism related genes in ΔBPSL3393 mutant versus wild type strain are shown in Figure 5. Interestingly, the expression of the *eut* genes and their putative transcriptional regulator were significantly down-regulated (*p* < 0.05) in ΔBPSL3393 mutant. This explained the reduced ethanolamine catabolism rate as indicated by the Biolog phenotype analysis. The molecular basis of this observation merits further investigation since BPSL3393 possibly acts as a CoA carrier protein participating in the ethanolamine metabolism. The transcripts of the *eut* genes were down-regulated by approximately 50% in the ΔBPSL3393 mutant compared to the wild type strain. In other words, the enzymes for ethanolamine metabolism were still available in the mutant although with lesser level. The level of enzymes was enough to support the mutant cell growth in ethanolamine medium although the growth rate was slower in minimal medium. Therefore, this explains the observation that no difference in growth rate between the ΔBPSL3393 mutant and wild type strains in the ethanolamine minimal medium.

## DISCUSSION

Hundreds of *B. pseudomallei* strains have been sequenced since its first complete genome sequence was made available (Holden *et al.* 2004). Genomes of *B. pseudomallei* comprise an approximately one third of uncharacterised hypothetical genes. This category of genes may possibly contribute to the unique intracellular survival of *B. pseudomallei* and thus unraveling their functions is virtually needed. In this study, a hypothetical gene BPSL3393 was chosen for functional validation using the reverse genetics approach. This gene putatively encodes a small protein of approximately 11 kDa in which deduced to be a CoA binding domain. It seemed to play a role in central metabolism that involves CoA moieties. CoA is a prominent compound since its derivatives are involved in many essential metabolic pathways. Thus, CoA-binding proteins are believed to have metabolic importance in all biological systems (Lipmann 1953, Engel & Wierenga 1996).

A putative promoter region −35 (ATGAAG) and −10 (CAAAAT) was detected at positions −123 bp and −143 bp relative to the putative initiation codon of BPSL3393. A putative ribosome binding site (RBS) with the sequence 5′-TACCT- 3′ was also found at approximately 8 bases upstream (5′ end) of the same start codon. This sequence and part of the 3′ end sequence (5′-AGGTA- 3′) of *B. pseudomallei* K96243 16S rRNA were found to be complementary. This complementarity is important to ensure the formation of a stable structure for translational initiation to occur (Shine & Dalgarno, 1974). The transcription of BPSL3393 was detected by using RNA microarray and a whole-genome transcriptome profiling as reported by Rodrigues *et al*. (2006) and Ooi *et al*. (2013), respectively. These data indicate that BPSL3393 is an active gene.

A strain carrying an unmarked deletion of BPSL3393 was constructed to study the function of this hypothetical gene. An analysis using the Biolog Phenotypic MicroArray system showed that this mutant demonstrated a significant reduction in the utilisation of 2-aminoethanol compared to the wild type. The compound 2-aminoethanol, or more commonly known as ethanolamine, is an important building precursor of phosphotidylethanolamine. Phosphotidylethanolamine and phosphotidylcholine are common precursors of the phospholipid bilayer component of cell membranes and the ratio of phosphatidylethanolamine to phosphatidylcholine influences membrane integrity (Li *et al.* 2006; Melchior 1982). However, both ΔBPSL3393 mutant and wild type strains showed similar cell outer morphology based on the SEM observations. Based on the TEM visualisation, both ΔBPSL3393 mutant and wild type strains also showed similar intracellular manifestation. Thus, the reduced utilisation of ethanolamine that resulted from the deletion of BPSL3393 did not cause any apparent alteration to the cell morphology. This observation also suggested that the localisation of ethanolamine permease was not altered upon mutation. Consequently, it was speculated that the ethanolamine metabolism in *B. pseudomallei* might contribute to other cellular functions instead of membrane biosynthesis.

The growth analysis indicated that both *B. pseudomallei* ΔBPSL3393 mutant and wild-type strains had similar growth pattern in the rich LB medium. Despite the reduction of ethanolamine assimilation, deletion of this gene in the mutant strain had minimal effect on its growth kinetic. A slower growth was observed for both strains in the M9 minimal medium. Their growth patterns were similar in the early stages of growth, the ΔBPSL3393 mutant cells self-aggregated after approximately 18 hours of incubation. This suggested that BPSL3393 might play a role in maintaining cell fitness in an elevated cell density in nutrient limited condition.

**Figure 6:**
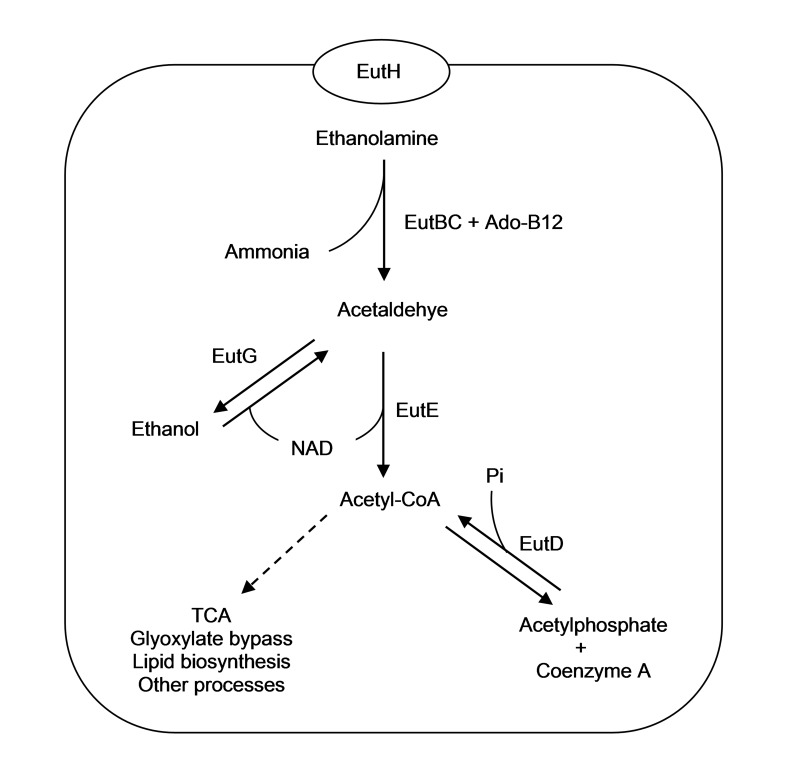
Ethanolamine catabolism (Stojiljkovic *et al.* 1995). Ethanolamine is first transported into cell with the help of EutH or diffusion. In the ethanolamine-specific microcompartment, ethanolamine is degraded to acetaldehyde and ammonia by the EutBC with cofactor Ado-B12. Acetaldehyde is either catabolised to ethanol by EutG or to the metabolically useful compound, acetyl-CoA, by EutE. Acetyl-CoA serves as initial subtract for various metabolic processes or converted into acetylphosphate by EutD. EutBC: Ethanolamine ammonia lyase; EutH: Ethanolamine utilization protein H; EutG: Alcohol dehydrogenase; EutE: Acetaldehyde dehydrogenase; EutD: Phosphotransacetylase; NAD: Nicotinamie Adenine Dinucleotide; Pi: Inorganic phosphate; TCA: Tricarboxylic acid cycle; Ado-B12: Adenosylcobalamin.

Ethanolamine can be utilised as the sole carbon and nitrogen sources in a wide variety of other bacteria such as *Pseudomonas*, *Salmonella*, *Klebsiella*, *Enterococcus*, *Mycobacterium*, *Clostridium* and *Escherichia*, especially those species that inhabit inside the mammalian gastrointestinal tract (Roof & Roth 1988; Blackwell *et al.* 1976). The best studied microorganism in ethanolamine utilization is *Salmonella* Typhimurium. The gastrointestinal tract contains a rich source of ethanolamine due to constant turnover and exfoliation of intestinal cells. In the bacterial cell, ethanolamine catabolism is executed inside a microcompartment known as carboxysome in order to retain and concentrate acetaldehyde. Acetaldehyde is a volatile intermediate which is potentially toxic to microorganisms and able to inhibit cell growth. Carboxysomes are consisted of structural proteins that encoded by *eutK*, *eutL*, *eutM*, *eutN* and *eutS* genes (Kofoid *et al.* 1999, Penrod & Roth 2006). As shown in Figure 6, the main enzymes involved in ethanolamine catabolism are EutH (ethanolamine permease), EutBC (ethanolamine ammonia lyase), EutE (acetaldehyde dehydrogenase) and EutG (alcohol dehydrogenase). EutH is responsible for ethanolamine uptake by cells. The imported ethanolamine is then degraded into ammonia and acetaldehyde by EutBC which is a coenzyme vitamin-B_12_ (5′-deoxyadenosyl cobalamin) dependent enzyme. Acetaldehyde is subsequently converted either to ethanol by EutG, or to acetyl-CoA by EutE (Garsin 2010). A transcriptional activator protein coded by *eutR* activates transcription of the *eut* operon in response to the simultaneous presence of B12 and ethanolamine (Roof & Roth 1992).

An analysis of the genome of *B. pseudomallei* K96243 revealed the presence of five *eut* homologs: BPSL3369 (*eutE*), BPSL3371 (*eutC*), BPSL3372 (*eutB*), BPSL3373 (*eutH*) and a putative transcriptional regulator BPSL3368 (*eutR*). However, the *eutG* homolog was not found and this gene product is responsible for conversion of acetaldehyde to ethanol. In *Salmonella* Typhimurium, mutations in *eutG* or *eutE* fully diminished the ability to utilise ethanolamine as a carbon source and reduced the ability to utilise ethanolamine as a nitrogen source (Stojiljkovic *et al.* 1995). Based on *S.* Typhimurium, the absence of EutG in *B. pseudomallei* should allow it to use ethanolamine as a nitrogen source but not as a carbon source. As shown in the growth studies using ethanolamine minimal media, the growth of *B. pseudomallei* was severely attenuated when grown in the ethanolamine media that with no extra carbon source. This result was in congruent with the observation of *S. Typhimurium.* The deletion also resulted in reduced transcriptions of the *eut* genes which are situated at a great distance from BPSL3393 within the same chromosome. However, a more comprehensive study is required to elucidate the transcriptional regulations of *eut* genes by BPSL3393. This reduction of the transcriptions explains the reduced ethanolamine utilisation rate in the mutant compared to the wild type strain.

The polypeptide of BPSL3393 is predicted to form a CoA binding domain which contains a Rossmann-fold structure. The Rossmann-fold is a common binding motif found in nucleotide-harboring coenzymes such as flavin adenine dinucleotide (FAD), nicotinamide adenine dinucleotides phosphate (NADP), nicotinamie adenine dinucleotide (NAD) and some CoA derivatives (Modis & Wierenga 1998; Rossman & Liljas 1974). Coincidentally, a conserved domain search analysis revealed that the Rossmann-fold was also present in EutC and EutE. The protein motifs of Rossmann-fold and CoA binding domain in BPSL3393 strongly suggested that this hypothetical protein might serve as a facilitator for EutE to combine the CoA and acetaldehyde to form acetyl-CoA. Since EutG is not present in *B. pseudomallei*, the conversion of acetaldehyde to ethanol is not possible and acetaldehyde could subsequently accumulate in the cell. Acetaldehyde is toxic and able to inhibit cell growth. However, the mutant cell growth did not attenuate as compared to the wild type strain when grown in ethanolamine supplemented minimal medium. This may be due to the presence of an alternative pathway that could accommodate the toxicity of acetaldehyde.

## CONCLUSION

BPSL3393 was found to be an auxiliary factor in ethanolamine catabolism and the deprivation of BPSL3393 down-regulated the *eut* genes. The transcriptional down-regulation of the ethanolamine genes hampered ethanolamine utilisation. It is likely that BPSL3393 and *eut* genes play a role in maintaining the cell fitness in challenging growth conditions since the mutant cells aggregated when it was grown in a poor medium.

## Figures and Tables

**Table 1:** Bacterial strains and plasmids.

Bacterial strains	Genotypes	Source/references
*Escherichia coli* S17-1 λpir	Tp^r^ Sm^r^ *rec*A *thi pro hsd*RM^+^ RP4-2-Tc::Mu-Km::Tn7*λpir*	Biomedal S.L.,Spain.
*Burkholderia pseudomallei* K96243	Wild type strain isolated from a 34 year old female at Khon Kaen Hospital	(Holden *et al.*, 2004)
*Burkholderia pseudomallei* K96243 ΔBPSL3393	Derivative of *B. pseudomallei* K96243 with unmarked deletion of gene BPSL3393	This study

Plasmids		

RBC TA cloning vector	TA cloning vector, *LacZ*, Amp^r^	RBC bioscience, USA
pUD-RBC	Derivative of RBC TA cloning vector containing the fused flanking region of gene BPSL3393	This study
pDM4	Suicidal plasmid, Cm^r^, *sacB* mobRP4, oriR6K	(Milton *et al.*, 1996)
pUD3393	Derivative of pDM4 vector containing the flanking region of gene BPSL3393	This study

**Table 2:** Primers used in mutant construction. The restriction sites are underlined.

Primer	Sequence (5′-3′)	Restriction site
USF	CTCGAGCGAAGAAGGCTTTTGCAACAT	*Xho*I
USR	CCTAGCATGCCATTTTGTCGCAGGTAAGCA	*Sph*I
DSF	CCTAGCATGCTAGCCCAGGCGCGTCGGCCGACA	*Sph*I
DSR	GAGCTCTATCGAGGAAACACGATGAAAATGAA	*Sac*I
USF-EXT	CGGTGATGCTCGGCTATT	-
DSR-EXT	CCGGCCGTCCGTTATGATCC	-
NQCAT	TAACGGCAAAAGCACCGCCGGACATCA	-
NQREV	ACATGTGGAATTGTGAGCGGATAACAA	-

**Table 3:** Primers designed for RT-qPCR study.

Primers	Sequence (5′-3′)	Genes
3368-F	GGCGATGCGTACTGGTT	AraC transcriptional regulator (*eutR*)
3368-R	GGCGTCAACAGCTCGAA	
3369-F	AGCATCTACGATCGCTTCATC	Acetaldehyde dehydrogenase (*eutE*)
3369-R	GTCGATGTACGACAGGATCTTC	
3371-F	AGCCTCGGCGTGTATCT	Ethanolamine ammnia-lyase light chain (*eutC*)
3371-R	GTGCGTCAGCAGGTAGTG	
3372-F	GATCAGGACGACATGGACAA	Ethanolamine ammonia-lyase heavy chain (*eutB*)
3372-R	GCTCTGGTAGTTCAGCATCA	
3373-F	CGGGATCATTCTCGGCTATTC	Ethanolamine permease (*eutP*)
3373-R	CGCGATAAGGCGTCTTGAA	
dnaK-F	CGCAGATCGAAGTGACCTT	Chaperone protein
dnaK-R	ATCTTCTCGATCTCGGCTTC	
